# Gastric Ischemia Secondary to Abdominal Distension

**DOI:** 10.7759/cureus.12793

**Published:** 2021-01-19

**Authors:** Sanjiv Gray, Andrew Hanna, Latha Ganti

**Affiliations:** 1 Surgery, University of Central Florida College of Medicine, Orlando, USA; 2 Emergency Medicine, University of Central Florida College of Medicine, Orlando, USA; 3 Emergency Medicine, Envision Physician Services, Plantation, USA; 4 Emergency Medicine, Osceola Regional Medical Center, Kissimmee, USA; 5 Emergency Medicine, HCA Healthcare Graduate Medical Education Consortium Emergency Medicine Residency Program of Greater Orlando, Olrando, USA

**Keywords:** gastric ischemia, atherosclerosis, sepsis, upper gastrointestinal bleeding

## Abstract

Gastric ischemia has been reported in the literature mainly as case reports and case series. It is uncommon because of its excellent blood supply. Patients with atherosclerosis and vascular insufficiency are at risk for gastric ischemia, especially with gastric distension. Workup may reveal radiographic findings that denote ischemia and would prompt surgical intervention. However, these patients with vascular insufficiency tend to be poor surgical candidates, which can present a dilemma. The authors report two cases where non-operative management for gastric ischemia was successfully accomplished.

## Introduction

Gastric ischemia is rare entity due the abundant vascular supply to the stomach. Indeed, there is a common surgical dictum that it is almost impossible to devascularize the stomach. Gastric ischemia can be seen in patients with acute gastric distension. As it is a rather rare entity, there is not much literature on the etiology, risk factors, or outcomes [1]. A handful of case studies have reported associations with volvulus [2], esophagectomy [3], invasive aspergillosis [4], critical stenosis of the celiac trunk [5], and abdominal distension. Computed tomography (CT) findings may include pneumatosis, perigastric venous gas, and hepatic portal system gas. The management can be conservative or surgical. We present two cases presenting with gastric ischemia secondary to acute distension that were managed conservatively with gastric decompression, broad-spectrum antibiotics, and acid suppression therapy.

## Case presentation

Case 1

A 69-year-old male smoker with a past medical history of hypertension, chronic obstructive pulmonary disease, hypothyroidism, peripheral vascular disease with history of multiple surgical interventions, superior mesenteric artery (SMA) stenosis, left carotid stenosis, dyslipidemia, coronary artery disease status post angioplasty and stent placement, chronic kidney disease, right lower extremity deep venous thrombosis, peptic ulcer disease, and ileostomy presented to the emergency department with complaints of constant upper abdominal pain for three days. The pain was associated with nausea, vomiting, and anorexia. He denied any blood or coffee grounds in the vomitus. He mentioned that the last time he urinated was one day ago. His vital signs revealed a temperature of 36.4 °C, blood pressure of 188/85 mmHg, pulse of 96 beats/minute, respiratory rate of 20 breaths/minute, and saturation of 96%. On physical examination, his abdomen was soft, non-distended, non-tender, and without guarding. Prior laparotomy scars were noted. The right upper quadrant ostomy was pink and viable. The patient had a past surgical history of left groin exploration with repair of the left inguinal hernia, exploratory laparotomy, resection of total colectomy and drainage of retroperitoneal abscess and debridement of the abdomen, end ileostomy, right lower extremity fasciotomy, left femoral-popliteal bypass graft, right lower extremity femoral-popliteal, and a right below the knee amputation.

**Figure 1:**
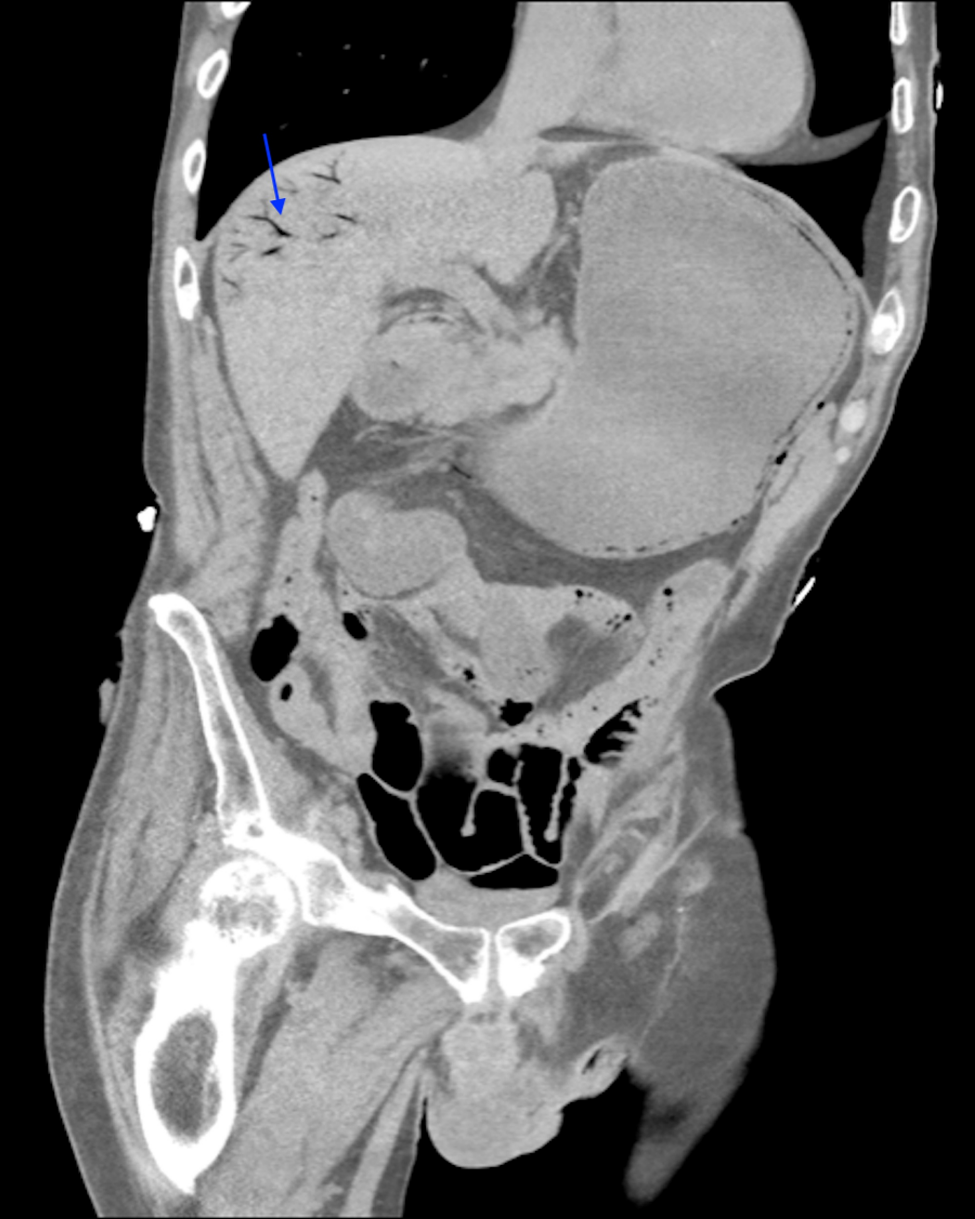
Coronal CT scan of the abdomen and pelvis demonstrating portal gas (blue arrow) and gastric outlet obstruction. CT, computed tomography

**Figure 2:**
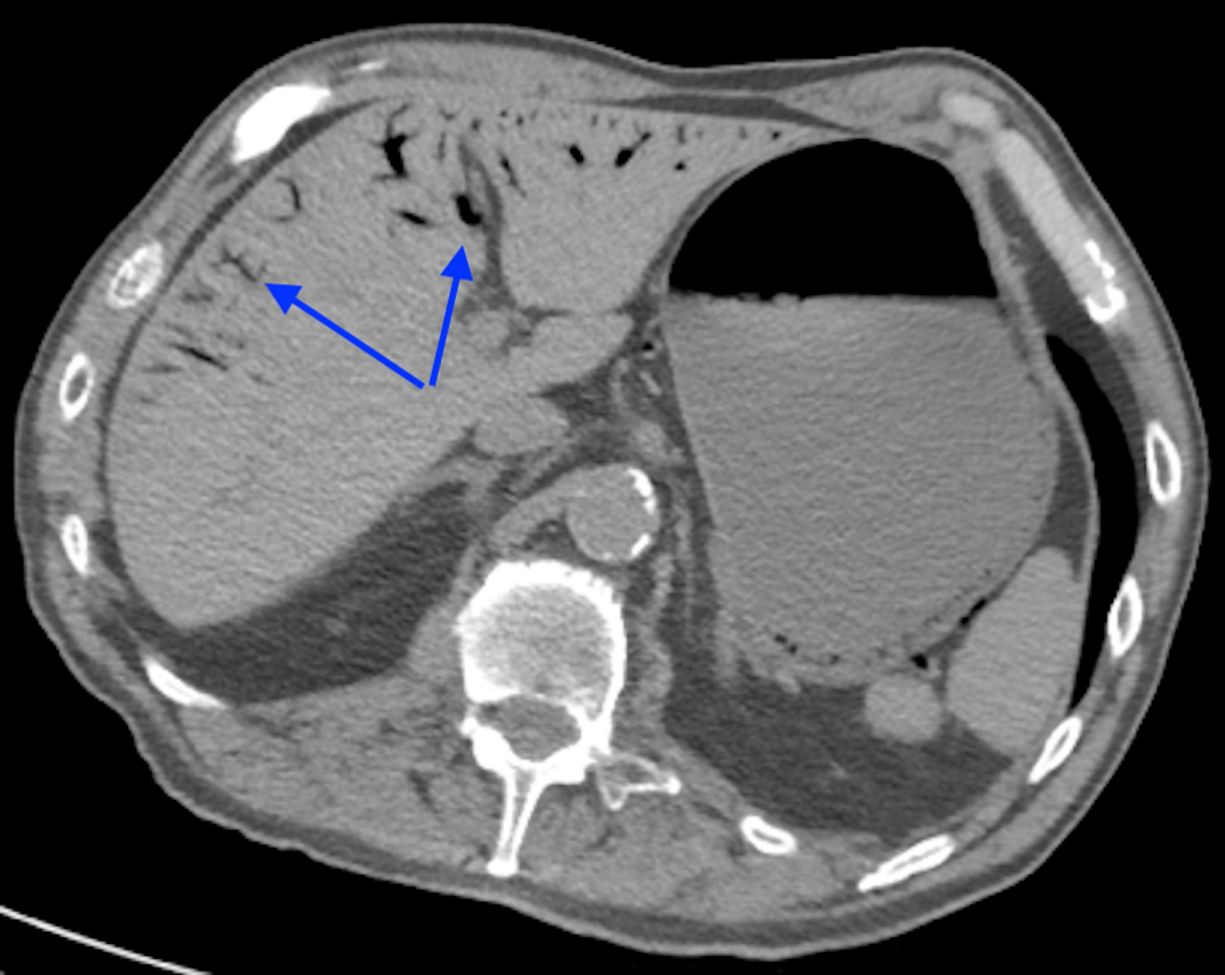
Axial CT scan of the abdomen demonstrating gas in the portal and perigastric veins (arrows). CT, computed tomography

Initial laboratory findings were significant for white blood cell (WBC) count of 21,000/μL, hematocrit 61.4%, hemoglobin 18.5 g/dL, platelet count 22,000/μL, chloride 116 mmol/L, glucose 227 mmol/L, blood urea nitrogen (BUN) 69 mg/dL, creatine 7 mg/dL, bicarbonate 8 mmol/L, lactic acid 7, pro B-type natriuretic peptide (BNP) 26,381 pg/mL, troponin 0.26 ng/mL, and procalcitonin 4.8 ng/mL. The patient was given broad-spectrum antibiotics and received 2 L of crystalloids. CT of the abdomen and pelvis revealed extensive portal venous gas seen throughout both hepatic lobes, small amount of air within the main portal vein, and perigastric veins with pneumatosis of the body of the stomach. There was distention of the stomach with minimal wall thickening. There was no free fluid, bowel dilatation, mesenteric edema or enhancement, or pneumatosis of the small bowel [Figures 1, 2].

**Figure 3:**
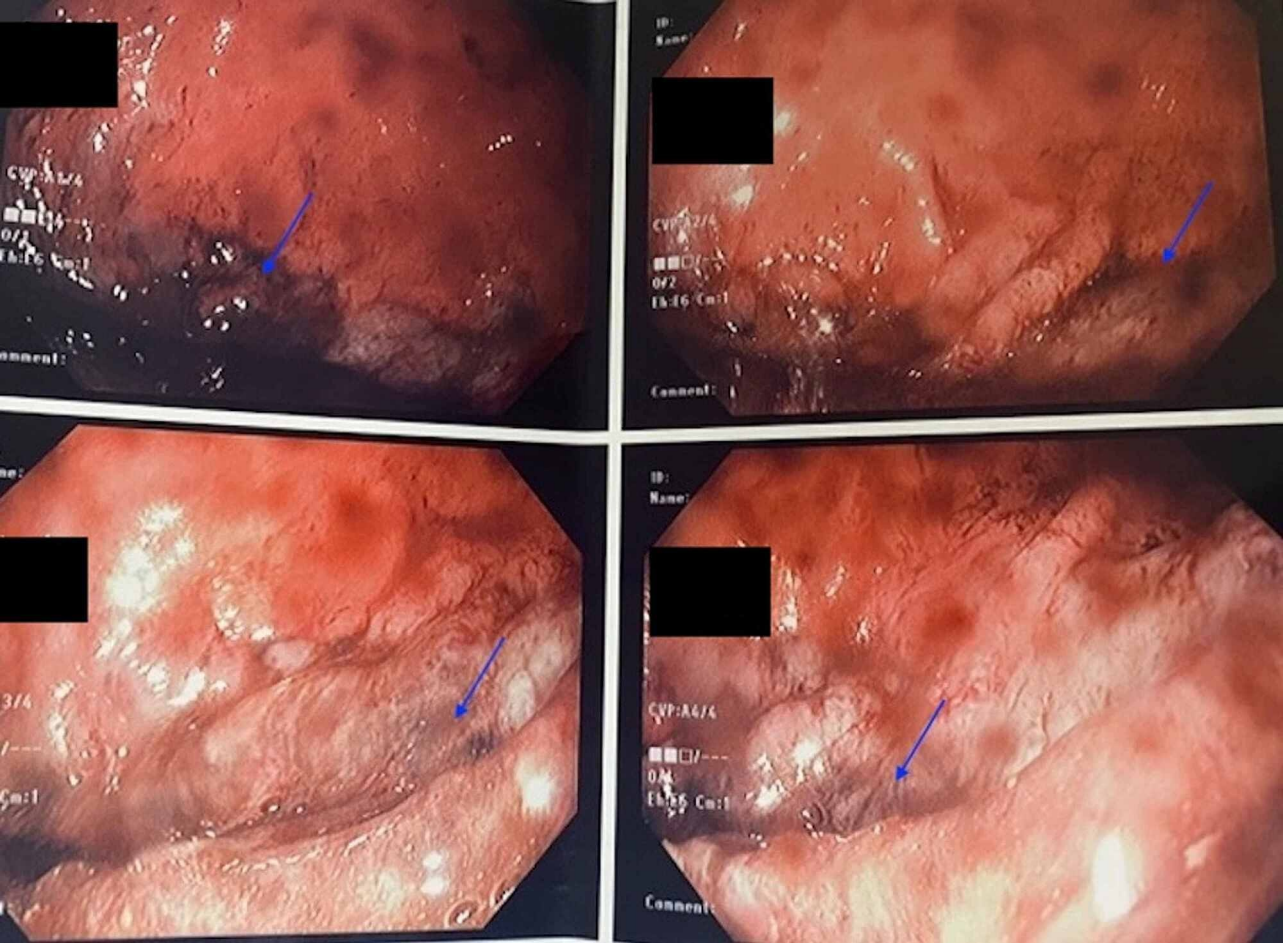
Endoscopy images revealing gastric ischemia (blue arrows).

The patient underwent upper endoscopy that showed severe distal Los Angeles class D erosive esophagitis, large amount of fluid retention in the stomach with some solid food along with evidence of localized ischemia in the greater curvature, and gastritis in the antrum. The pylorus was almost completely shutdown with the scope being able to advance beyond the pylorus [Figure 3].

On day two in the hospital, the patient denied abdominal pain, nausea, or vomiting. He was on low-dose norepinephrine to maintain his mean arterial pressure above 65 mmHg. He remained oliguric but cleared his lactate. His WBC decreased to 10,000/μL. On the third day, he was transferred out of the intensive care unit (ICU) with reduced nasogastric (NG) aspirate, which was now clear. His abdomen remained soft and non-tender. On the fifth day, upper gastrointestinal series was done and showed contrast flowing into the small intestines. The NG tube was removed and the patient was started on a clear liquid diet. On the seventh day, he had improved renal function evidenced by adequate urine output and increased glomerular filtration rate. A mesenteric angiogram was done via the right common femoral artery that demonstrated approximately 75% stenosis at the origin of the SMA and approximately 75% stenosis at the origin of the celiac artery. Angioplasty and stenting of the SMA was done using a 5 × 4 mm balloon and a 7 × 29 mm stent. On the tenth day, the patient was discharged home tolerating a mechanical soft diet with supplemental protein shakes.

Case 2

**Figure 4:**
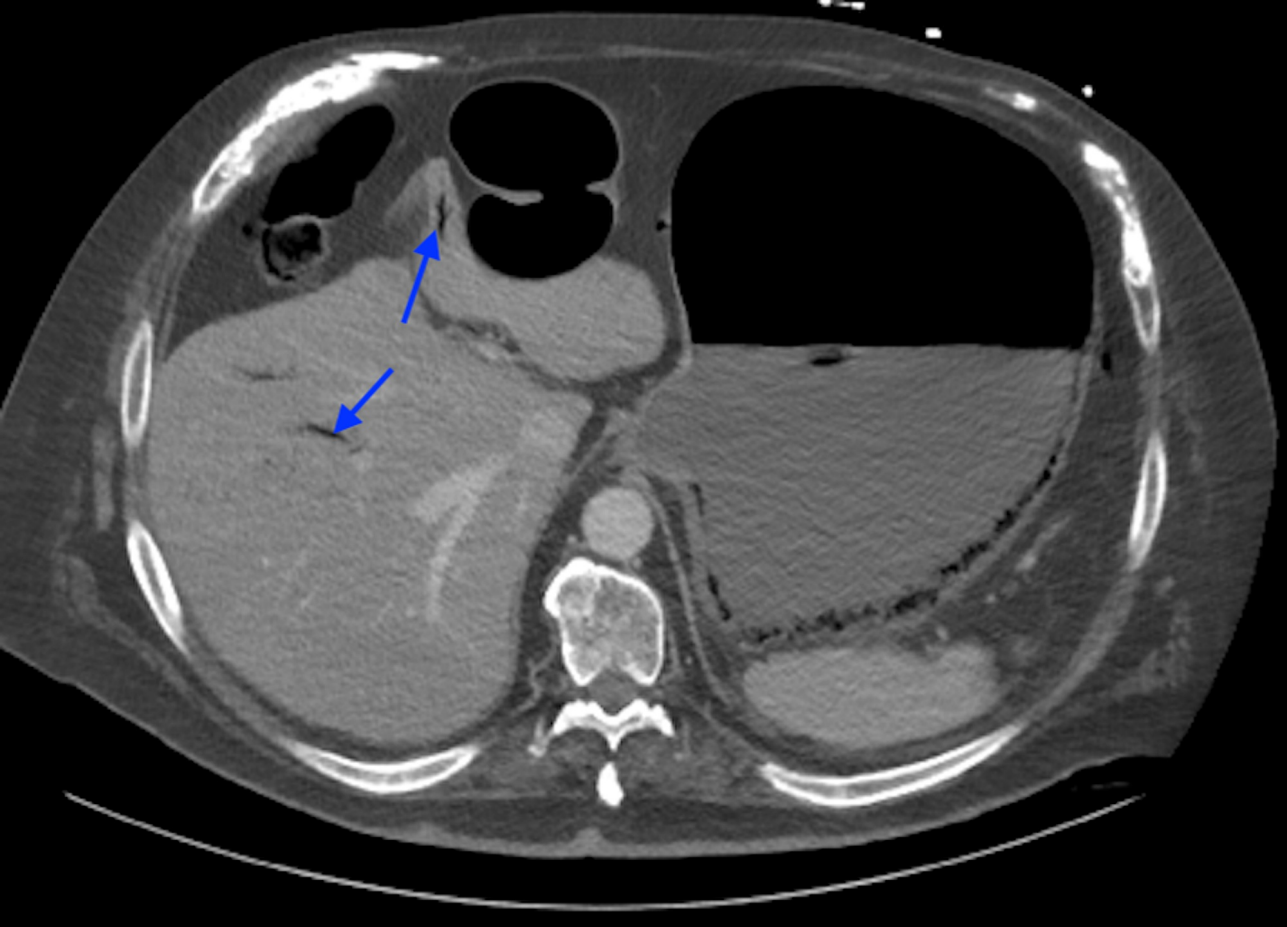
Axial CT of the abdomen demonstrating gas in the portal and perigastric veins (arrows). CT, computed tomography

A 65-year-old male with past medical history of diabetes mellitus, deep venous thrombosis on apixaban, myocardial infarction, hypertension, atrial fibrillation, hypoalbuminemia, emphysema, and morbid obesity presented to the emergency department from a nursing home. The patient complained of abdominal pain, nausea, vomiting, and diarrhea for 24 hours. The patient mentioned an inability to tolerate solid food for approximately one year. The physical examination showed no acute distress, no respiratory distress, atrial fibrillation with rapid ventricular response, and the abdomen was obese and non-tender, without rebound or guarding. An NG tube was placed. His vital signs revealed a temperature of 36.5 °C, blood pressure 143/119 mmHg , pulse 163 beats/minute, respiratory rate 19 breaths/minute, and oxygen saturation 99%. Laboratory evaluation showed WBC count of 26 k/μL with 90% neutrophils, albumin 1.4 mmol/L, BNP 2,300 pg/mL, international normalized ratio 1.4, BUN 22 mg/dL, and creatinine 1.1 mg/dL. A CT scan of the abdomen and pelvis with intravenous contrast showed severe distention of the stomach and duodenum with extensive gas in the wall of the dependent portion of the stomach and extension of gas into the adjacent gastric veins, portal vein, and portal vein branches within the liver [Figure 4].

The branches of the celiac artery and SMA were patent. The patient was resuscitated with crystalloids, given broad-spectrum antibiotics, NG tube for gastric decompression, diltiazem infusion for atrial fibrillation, and admitted to the ICU. The patient declined any surgical intervention.

**Figure 5:**
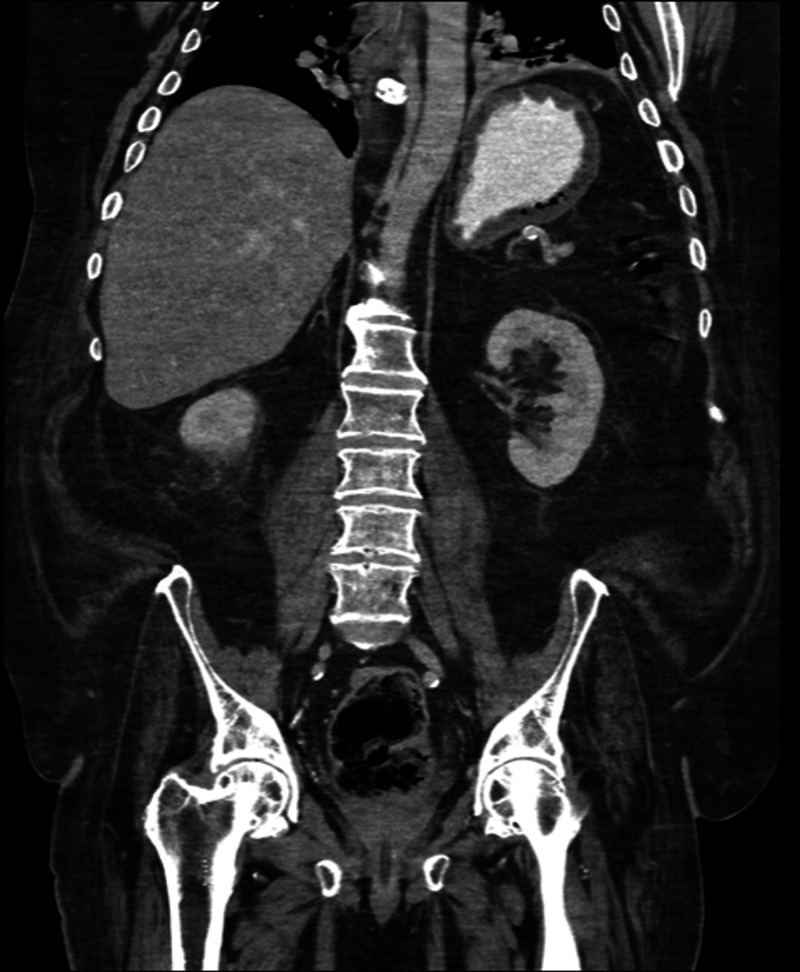
Coronal CT scan of the abdomen demonstrating resolution of air in the left gastric vein and the gastroepiploic veins. CT, computed tomography

On the second day in the hospital, the patient showed improvement with his abdominal pain, now a 4/10 down from 10/10 on admission. The patient remained on a diltiazem drip for his atrial fibrillation with rapid ventricular response. His abdomen was soft and non-tender; however, his NG tube output was approximately 1 L of bilious fluid. On the third day, the NG tube output was 900 mL of bilious fluid overnight and he had no bowel movement since admission. The WBC reduced to 12,000/μL and he had a benign abdominal examination. CT scan was done with enteral and intravenous contrast to assess for bowel obstruction. It showed that the stomach was now decompressed and there was decreased air within the wall of the stomach and the previously seen air within the left gastric vein and the gastroepiploic veins had resolved [Figure 5].

The small intestines, appendix, and colon appeared normal. The patient’s diet was progressed as he tolerated and he was discharged back to the skilled nursing facility on the tenth day.

## Discussion

Gastric ischemia is a rare finding that can be seen in patients with sepsis, severe atherosclerosis, vasculitis, acute gastric dilatation, gastric volvulus, or due to idiopathic causes [6]. The stomach has a rich vascular supply from the left gastric, right gastric, right, and left gastroepiploic arteries and the short gastrics and two arterial arcades, as well as several collateral sources. Gastric ischemia is associated with 30-40% mortality [6-8]. A multicenter case series of 12 gastric ischemia patients reported the common presentation of abdominal pain, nausea, and symptomatic anemia [7]. Patients may also present with vomiting, weight loss, anorexia, and gastrointestinal bleeding [8,9]. The underlying etiology may be post angioembolization, postoperative including endoscopy, hypotension in patient with preexisting celiac stenosis, vasculitis, acute gastric dilatation, volvulus, necrotizing gastritis, and caustic ingestions [7-10]. Risk factors include smoking, atherosclerosis, diabetes mellitus, advance age, and hypertension. All these risk factors increase the risk for vasculopathy and gastropathy in general and may be synergic for gastric ischemia [10]. While any portion of the stomach can be affected, the greater curvature and the posterior wall of the body and fundus are the most impacted [9,10]. The diagnostic workup involves a CT scan of the abdomen. Co-existing renal insufficiency may prevent the administration of intravenous contrast. Common CT findings include pneumatosis, perigastric venous gas, and hepatic portal system gas. CT scan may also reveal gastric dilatation, gastric outlet obstruction, volvulus, focal ulceration, gastric wall thickening, and mesenteric vascular diseases such as atherosclerosis, stenosis, vasculitis, and thrombosis. A negative CT scan does not rule out gastric ischemia [10]. CT angiography or catheter-based angiography can be done after adequate resuscitation to reduce the risk of renal insults. Endoscopy can be diagnostic and therapeutic, especially for patients with volvulus, and help with the decision of surgical intervention. Endoscopy is deemed to be safe even in the presence of gastric pneumatosis [9]. It is debatable whether gastric ischemia causes gastric dilatation or whether gastric dilatation causes the ischemia. Common findings are mucosal congestion, erythematous and pale changes, ulceration, gastritis, and necrosis [6,8-10]. Histopathological changes can be along the spectrum ranging from capillary dilatation, mucosal edema, vascular congestion or superficial surface erosions, hyalinization and fibrosis of lamina propria, hemorrhagic gastritis, and withered atrophic glandular epithelium [8,9].

Management involves prompt gastric decompression via an NG tube, acid suppression therapy, and broad-spectrum antibiotics as a part of the general sepsis resuscitation and to cover for possible emphysematous gastritis. Surgical intervention is required for patients with peritonitis, free perforation, extensive necrosis, obstruction, bleeding, failed conservative management, and those requiring mesenteric revascularization [5,10]. The surgical management is complex and may require partial versus total gastrectomy, with or without enteral anastomosis, and revascularization if indicated. Damage control should be used in patients in extremis and tube feeding should be considered for patients requiring total gastrectomy. Follow-up imaging and endoscopy maybe required for adequacy of treatment and management of the underlying etiology. A retrospective review of 17 patients detailing the natural history of the disease showed a 24% mortality at six months [10].

The cases described in this report show the common presentation of acute abdominal pain and gastric dilatation. CT scans were significant for pneumatosis of stomach, and both patients were managed conservatively with improvement in symptoms within a few days. Follow-up imaging showed resolution of the pneumatosis and dilatation, and both patients were discharged home on an enteral diet.

## Conclusions

Gastric ischemia is a rare clinical entity. The management is based on the clinical status of the patient and not solely on radiographic findings. Non-operative management can be pursued in appropriate patients without peritonitis and involves gastric decompression, broad-spectrum antibiotics, and acid suppression. Prospective studies are needed to assess the long-term efficacy and outcome of conservative versus surgical intervention, recurrence, and mortality outcomes.
